# Isolation and Partial Characterization of an Antifungal Protein Produced by *Bacillus licheniformis* BS-3

**DOI:** 10.3390/molecules17067336

**Published:** 2012-06-14

**Authors:** Tang-Bing Cui, Hai-Yun Chai, Li-Xiang Jiang

**Affiliations:** School of Biological Science and Bioengineering, South China University of Technology, Guangzhou 510006, China; Email: chaihaiyun-34@163.com (H.-Y.C.); 578792824@qq.com (L.-X.J.)

**Keywords:** *Bacillus licheniformis*, antifungal protein, chromatography isolation, proteinase

## Abstract

An antifungal protein produced by *Bacillus licheniformis* strain BS-3 was purified to homogeneity by ammonium sulfate precipitation, DEAE-52 column chromatography and Sephadex G-75 column chromatography. The purified protein was designated as F2 protein, inhibited the growth of *Aspergillus niger*, *Magnaporthe oryzae* and *Rhizoctonia solani*. F2 protein was a monomer with approximately molecular weight of 31 kDa in sodium dodecyl sulfate-polyacrylamide gel electrophoresis and gave a single peak on High Performance Liquid Chromatography (HPLC). Using *Rhizoctonia solani* as the indicator strain, the EC_50_ of F2 protein was 35.82 µg/mL, displaying a higher antifungal activity in a range of pH 6.0 to pH 10.0, and at a temperature below 70 °C for 30 min. F2 protein was moderately resistant to hydrolysis by trypsin, proteinase K, after which its relative activities were 41.7% and 59.5%, respectively. F2 protein was assayed using various substrates to determine the enzymatic activities, the results showed the hydrolyzing activity on casein, however, no enzymatic activities on colloidal chitin, CM-cellulose, xylan, *M. lysodeikticus*, and *p*-nitrophenyl-*N*-acetylglucosaminide.

## 1. Introduction

*Bacillus licheniformis* is a saprophytic bacterium that is widespread in Nature. It has been applied widely to produce proteases [1], amylases [2], lactamase [3], antibiotics [4], surfactin [5] and special chemicals [6] in the fermentation industry with low risk of adverse effects to human health or the environment. Bacitracin, the first peptide antibiotic derived from cultures of *B. licheniformis*, has been applied widely in the medical and veterinary area, and several other peptide antibiotics from different *B. licheniformis* strains have been studied [7].

Recently, three bacteriocin-like peptides named lichenin, bacillocin 490 and P40 produced by *B. licheniformis* strain 26 L-10/3RA, 490/5 and P40, respectively, have been reported [1,8,9]. These three bacteriocin-like peptides have different molecular mass, heat resistance, antagonistic spectrum and insensitivity to trypsin, and showed antagonistic activity against *Staphylococcusaureus*, and fungal species. A low-molecular-weight bacteriocin-like protein from *B. licheniformis* MKU3 exhibited a wide spectrum of antimicrobial activity against several Gram-positive bacteria, several fungi and yeast. A 3.6-fold increase in the production of bacteriocin is achieved using the culture medium optimized through a fractional factorial design [10]. *B. licheniformis* ZJU12 is isolated from soil, its cell-free supernatant exhibites marked antibacterial and antifungal activities, after treatment with proteinase K and trypsin, The antagonistic activity is lost completely by treating with proteinase K and trypsin [11]. In this paper, we introduced the isolation and partial characterization of a 31 kDa antifungal protein produced by *B. licheniformis* BS-3.

## 2. Results

### 2.1. Purification of the Antifungal Protein

**Figure 1:**
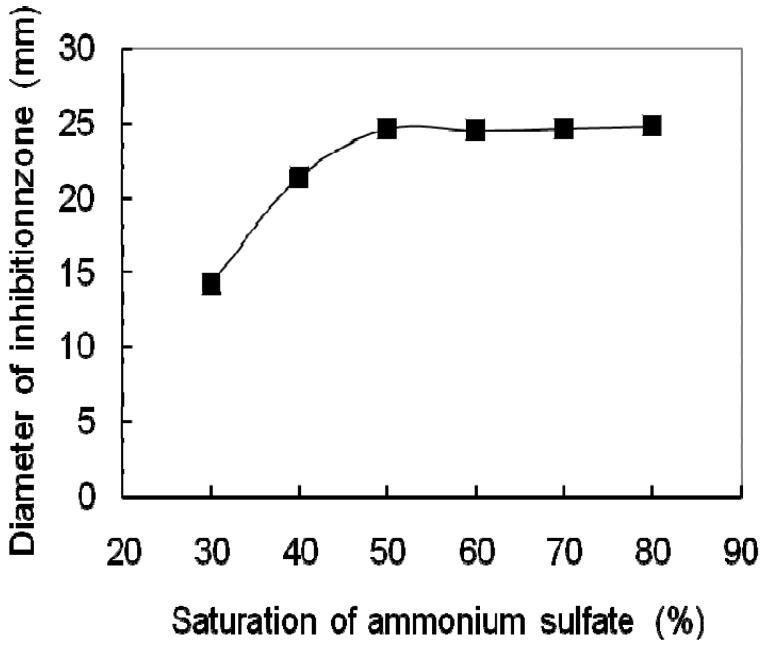
The effect of saturations of ammonia sulfate on the antifungal protein precipitation of BS-3.

The culture supernatant of BS-3 was filtered by filter-sterilizer. 600 mL of the filtrate was divided into six aliquots, precipitated with solid ammonium sulfate, The results showed that the optimal saturation of ammonium sulfate was 50% for precipitating antifungal protein (Figure 1).

**Figure 2:**
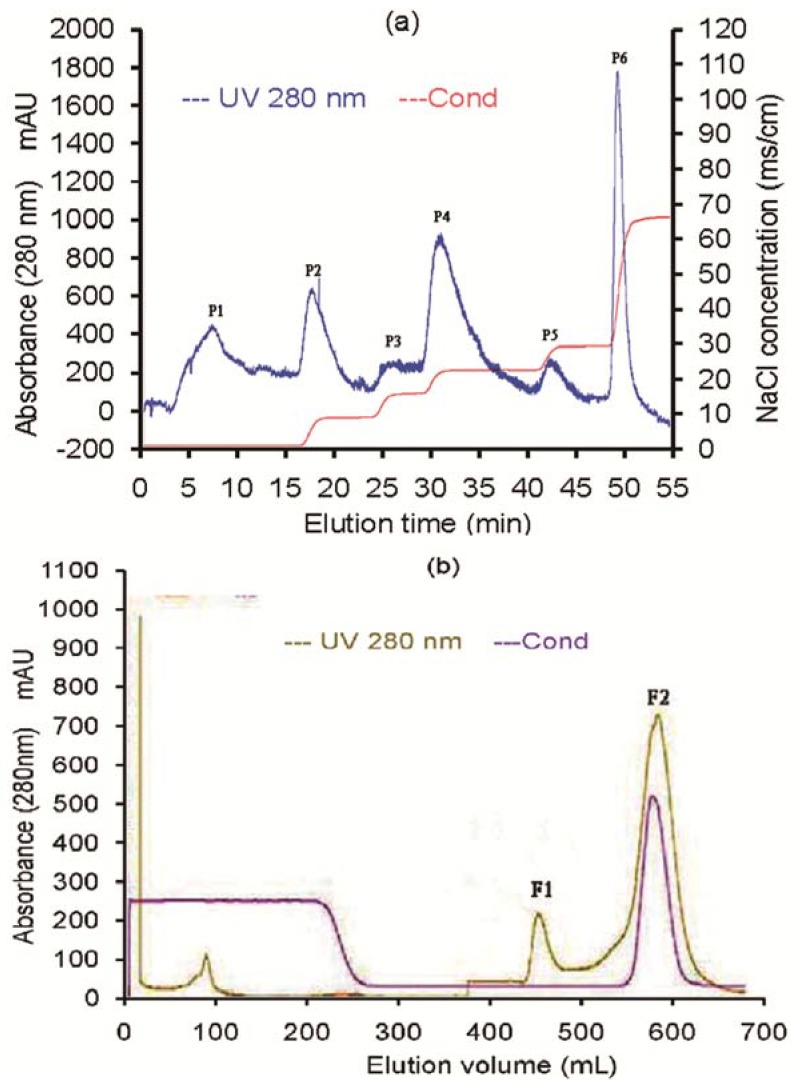
Purification of the antifungal protein (**a**) DEAE cation chromatography of crude extract from strain BS-3. The column was washed with 0.2 mol/L sodium acetate buffer (pH 5.6) to remove unadsorbed fractions (P1), and then eluted with 0.5 mol/L NaCl gradient (20%-100%) in the same buffer at a rate of 2.5 mL/min to obtain five fractions (P2, P3, P4, P5 and P6); (**b**) The P4 fraction in A was subjected to gel filtration chromatography on a Sephadex G-75 column. The column was eluted with buffer B (20 mol/L PBS buffer, pH 7.4), at a rate of 2 mL/min.

**Figure 3:**
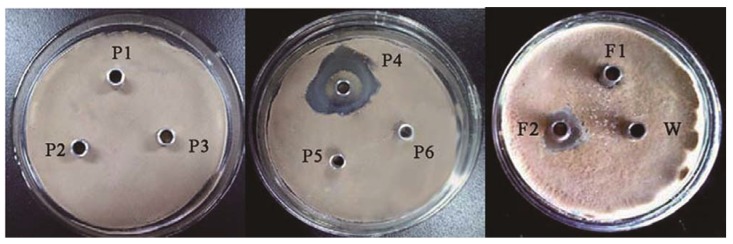
Antifungal activity of fractions separated by column chromatography P1-P6 fractions were separated by DEAE cation chromatography, F1 and F2 fractions were separated by G-75 gel filtration chromatography, W was sterile water.

To enrich antifungal protein from the culture of *B. licheniformis* BS-3, we first applied the crude extracts to cation exchange chromatography on a DEAE-52 column, which produced six fractions (Figure 2a). The six fractions were assayed for growth inhibition on the test fungus, *Rhizoctonia solani*. No antifungal activity was detected in the P1, P2, P3, P5 and P6, but antifungal activity was found in the P4 resulting from a linear NaCl gradient elution (Figure 3). Further purification for the P4 was conducted through the gel filtration chromatography on a Sephadex G-75 column and two main peaks were obtained (Figure 2b).

**Figure 4:**
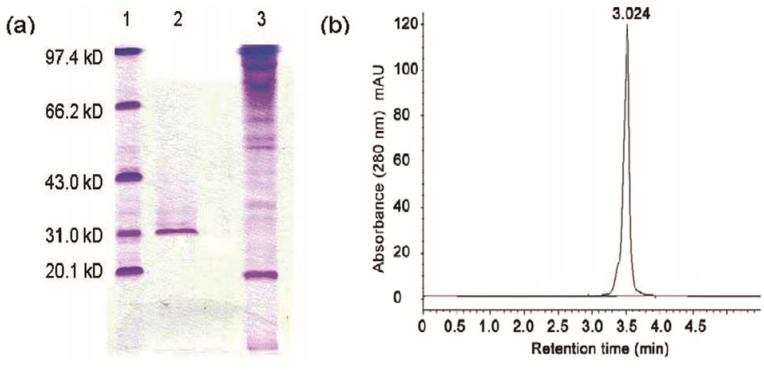
SDS-polyacrylamide gel electrophoresis and HPLC of F2 protein (**a**) F2 protein was analyzed by SDS-PAGE on 12% separating gel with a 5% stacking gel stained withCoomassie brilliant blue R-250 (Amresco). Lane 1, molecular mass marker proteins from Bio-Rad. Lane 2 and 3 were the F2 fraction and F1 fraction respectively from gel filtration chromatography on a Sephadex G-75 column; (**b**) HPLC chromatogram of F2 fraction.

After each peak was assayed for the antifungal activity to *Rhizoctonia solani*, we found that the second fraction (F2, Figure 3) showed antifungal activities. This antifungal protein was purified to homogeneity as determined by SDS-PAGE and RP-HPLC (retention time of 3.024 min), and its relative molecular mass was about 31 kDa (Figure 4). We designated this antifungal protein as F2 protein. From 1,000 mL culture supernatant of *B. licheniformis* BS-3, 74.31 mg purified F2 protein was obtained (Table 1).

**Table 1:** Summary for purification of antifugal protein from the culture supernatant of *B. licheniformis* BS-3.

Step	Amount of proteins (mg)	Total activity (AU)	Specific activity (AU/mg)	Recovery (%)	Purification (-fold)
**Crude supernatant**	747.24	53,770	71.96	100.00	1.00
**Ammonium sulfate precipitation**	186.86	50,482	270.16	93.89	3.75
**DEAE cation exchange**	113.67	43,940	386.56	81.72	5.40
**Gel filtration chromatography**	74.31	23,200	1352.85	43.15	18.80

### 2.2. Inhibitory Spectrum

F2 protein was tested for the presence of antifungal activity against five strains including *Aspergillus niger*, *Magnaporthe oryzae*, *Rhizoctonia solani*, *Fusarium oxysporum *(schl.) f. sp. *benincasae* and *Fusarium oxysporum *(schl.) f. sp. *momordicae*. The diameter of the inhibition zones for these strains was 25.0, 27.5, 20.3, 0.0 and 10.3 mm in the above order. Using *Rhizoctonia solani* as the indicator strain, the EC_50_ of F2 protein was 35.82 µg/mL.

### 2.3. Effects of Enzymes, Heat, and pH

**Figure 5:**
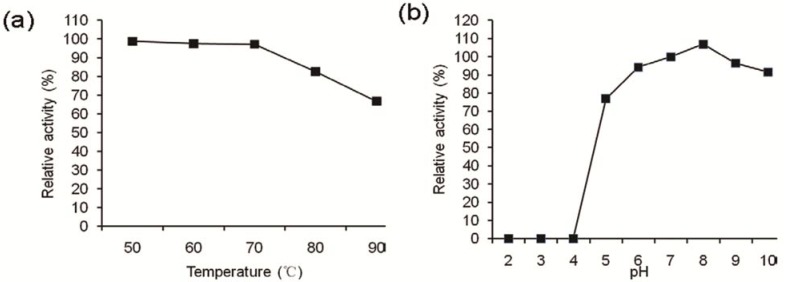
Effect of pH and temperature on the antifugal activity of the purified protein (**a**) The effect of temperature was determined by incubating the F2 protein at different temperatures for 30 min; (**b**) The effect of pH was determined by incubating the F2 protein at different pH for 30 min. The residual antifungal activity was estimated as described in the Experimental.

F2 protein was moderately sensitive to trypsin and proteinase K, and after treatment by these two enzymes, the relative activities were 41.7% and 59.5% respectively. For thermal stability determination, F2 protein in buffer A was incubated at different temperatures for 30 min, respectively. After cooling to room temperature, the residual antifungal activities were measured as described. F2 protein displayed higher antifungal activity, retaining more than 97% activity at temperatures below 70 °C, while at temperatures above 70 °C, the decrease in antifungal activity was gradual and the activity at 90 °C was only 66.8% of that at 70 °C (Figure 5a).

F2 protein was not active under acid conditions rom pH 2 to pH 4; however, its activities were higher and all exceeded 90% under pH 6 to pH 10 conditions. The optimal pH for antifungal activity of F2 protein was pH 8.0 (Figure 5b). Results showed that the antifungal activity was stable in neutrality and alkaline conditions for the related applications in the future.

### 2.4. Enzymatic Activities

The F2 protein solution was assayed using various substrates to determine the enzymatic activities, the results showed that the hydrolyzing activity on casein was 5,140 U/mL, however, it displayed no enzymatic activities on colloidal chitin, CM-cellulose, xylan, *M. lysodeikticus*, *p*-nitrophenyl-*N*-acetylglucosaminide (Table 2).

**Table 2:** Enzyme activities shown by F2 protein solution determined using various substrates.

Enzymes	Substrates	Concentration	Enzyme activity (U/mL)
Chitinase	Colloidal chitin	1%	n.d.
Cellulase	CM-cellulose	1.25%	n.d.
Xylanase	Xylan	0.5%	n.d.
Protease	Casein	1%	5,140
Lyozyme	*M. lysodeikticus*	Optical density of 1.5	n.d.
β- *N*-acetyl-glucosaminidase	*p*-Nitrophenyl-*N*-acetylglucosaminide	5 mM	n.d.

n.d.: Not detectable.

## 3. Discussion

Bacillus species have been used since many years for insect biocontrol, industrial enzyme production and antibiotic production. Biological control has been shown to be effective in many crops. Biological control using natural antagonistic microorganisms has been extensively studied. Many strains of the genus *Bacillus *have received much attention as biological control agents because of their advantages over Gram-negative bacteria and fungal biological control agents [12,13]. Several strains of *Bacillus licheniformis* species have been successfully used for biocontrol of plant pathogenic fungi, such as Botrytis *cinerea* and *Phytophthora capsici* [14,15,16].

Export of multiple substances from the cytoplasm to the extracellular environment is a common phenomenon for all kinds of cells, including bacteria. Exported bacterial substances can fulfill different functions, *B. licheniformis *is known to secrete a number of different substances into the extracellular medium and this ability has been used in the fermentation industry for a long time. Many of these substances have also antifungal activity, such as peptide [15,17], biosurfactant [5,18], Chitinase [19], Laccase [20]. Many bacteriocins from Gram-positive bacteria, some of which are produced by *B. licheniformis* strains, have been isolated from buffalo rumen and the Amazon basin [1,8].

Our study revealed that the antifungal substance in the culture of the B. *licheniformis* BS-3 was a protein. The cell-free supernatant obtained from the B. *licheniformis* BS-3 was purified by the three-step purification method. The purified protein was identified to homogeneity by SDS-PAGE and HPLC, it was designated F2 Protein. F2 protein strongly inhibited growth of several fungi including *Aspergillus niger*, *Magnaporthe oryzae, Rhizoctonia solani* and *Fusarium oxysporum (schl.)* f. sp. momordicae. SDS-PAGE analysis of the antifungal F2 protein revealed that its relative molecular mass was approximately 31 kDa. The molecular weight of F2 protein, differed clearly from other antifungal agents (enzymes, peptides and proteins) produced by B. *licheniformis*. B. *licheniformis* produces various antifungal substances with molecular masses ranging from 1.4 to 20 kDa [1,5,9,10,11,15,17,21].

It has been reported that several proteinase has antifungal activity [22,23,24]. We proved that F2 protein was a proteinase, it was moderately sensitive to proteinase K and trypsin, and had optimum antifungal activity in pH 8.0 and 50 °C. The results obtained for F2 protein did not accord with characteristics of the antifungal protein bacisubin and other antifungal proteins reported so far from *B*. *licheniformis*. To compare the structure difference between F2 protein and other antifungal proteases, proteases and antifungal proteins, we tried to analyze the *N*-terminal sequence of F2 protein. It was found that the *N*-terminus of F2 protein is blocked. From molecular mass and characteristics of F2 protein, it seems to be similar to keratinase produced by *B. licheniformis KI8102 *[25], and a subtilisin-like alkaline serine proteinase from B. licheniformis RKK-04 [26]. The purified keratinase has a molecular mass of 32 kDa and its optimum temperature and pH are 50 °C and 7.5, respectively. The alkaline serine proteinase has a molecular mass of 31 kDa and its optimum pH and temperature are found to be 10.0 and 50°C, respectively, but it is unclear if these two enzymes have antifungal activity.

## 4. Experimental

### 4.1. Antifungal Strain

An organism strain, showing marked antifungal activities, was isolated from soil of the South China University of Technology Campus (Guangzhou, China). Based on the 16S rRNA sequence, this strain was characterized as *B. licheniformis* BS-3 (accession No. JN867130). This strain was preserved in agar slant medium (containing potato 200 g/L, beef extract 5 g/L, glucose 20 g/L, pH 7.0, agar 20 g/L) at 4 °C. The seed culture was grown in liquid medium (containing beef extract 8 g/L, yeast extract 5 g/L, glucose 10 g/L, pH 7.0, agar 20 g/L) at 30 °C for 20 h.

### 4.2. Indicator Strains

*Rhizoctonia solani*, *Aspergillus niger*, *Magnaporthe oryzae*, *andFusarium oxysporum *(schl.) f. sp. *benincasae* were supplied by Xie Dasen from the Guangdong Academy of Agricultural Sciences (Guangzhou, China). The fungal strains were grown in PDA agar plate medium (containing potato 200 g/L, glucose 20 g/L, agar 20 g/L, pH 7.0) at 28 °C.

### 4.3. Growth and Antifungal Protein Production

*B. licheniformis *BS-3 was grown in three 300 mL triangular flasks with 50 mL liquid medium (containing glucose 15.0 g/L, peptone 5.0 g/L, MgSO_4_·7H_2_O 0.7 g/L, NaH_2_PO_4_·2H_2_O 2.0 g/L, Na_2_HPO_4_·2H_2_O 4.0 g/L, Tween-80 1 g/L, pH 7.5) each for 48 h at 28 °C with constant agitation. The average antifungal activity (three parallel tests) using *Rhizoctonia solani* as the indicator.

### 4.4. Quantification of Antifungal Activity

Using *Rhizoctonia solani* as the indicator, the antifungal activity of the sample was tested by the cylinder-plate method. The indicator was taken one loop by inoculating loop, added into a test tube containing 5 mL sterile water, indicating fungus suspension was oscillated for 12 h on a shaker. Indicating fungus suspension (100 μL) was taken out and spreaded evenly on PDA media plate. After 15 min, Oxford-cups were placed on the plate according to equal intervals, sample (100 µL) which by two-fold serial dilutions was added into Oxford-cups, then the plate was placed in the incubator, and cultured for 3 d at 30 °C. The antifungal activities of the sample for other three indicator strains were tested according to the same as above. The antifungal activity was expressed in terms of arbitrary units (AU). It was defined as the maximum dilution which produced a minimum of zone that still gave a clearly visible antagonistic zone. The reciprocal of the dilution gave the titer of antagonistic activity in AU per milliliter.

To determine the EC_50_ value (the concentration of the antifungal protein required to reduce the area of the mycelial colony to 50%) for the antifungal activity of F2 protein against *Rhizoctonia solani*, five doses (0.02, 0.04, 0.08, 0.16 and 0.32 mg) of the protein dissolved in phosphate buffer (pH 7.0) were mixed with each 10 mL of PDA at 45 °C, respectively. Petri dishes (9 cm diameter) were inoculated with *Rhizoctonia solani*-grown agar disks and incubated at 30 °C. Buffer only (without protein) served as control. For comparison a dilution series of the culture filtrate was included in this experiment. When the mycelium of the control had reached the edge of the plate, the areas of the mycelium colonies of the different treatments were measured, and the EC50 values were determined.

### 4.5. Extraction and Purification of Antifungal Protein

The culture supernatant of BS-3 was obtained by centrifugation at 6,000 r/min and 4 °C for 20 min, and then filtered by filter-sterilizer (aperture 0.22 μm, Millipore). The filtrate was precipitated with solid ammonium sulfate at different saturation overnight at 4 °C. The resulting precipitate was harvested by centrifugation at 6,000 r/min for 30 min, then redissolved in buffer A (0.2 mol/L sodium acetate buffer, pH 5.6), the solution was dialyzed against the same buffer for 24 h. The crude extract was passed through a DEAE-52 column (2.6 × 50 cm) on an AKTA Prime system (Amersham Biosciences, Uppsala, Sweden) pre-equilibrated with buffer A, and then the column was washed with the same buffer to remove unabsorbed proteins. The adsorbed proteins were eluted with a linear gradient of 0.5 mol/L NaCl from 20% to100% concentration in buffer A at a flow rate of 2.5 mL/min. Antifungal activity against *Rhizoctonia solani* was monitored in each fraction. The antifungal fractions were pooled, and then applied to a Sephadex G-75 column (2.6 × 50 cm) on an AKTA Prime system (Amersham Biosciences) pre-equilibrated with buffer B (20 mmol/L PBS buffer, pH 7.4), the column was eluted with buffer B at a flow rate of 2.0 mL/min. Individual peak fractions were collected and condensed in a cut-off dialysis tubing (Sigma). After dialysis against ultrapurified water, the samples were used for further analysis. All purification steps were performed at room temperature, and the column effluent was monitored by absorbance at 280 nm.

### 4.6. Electrophoresis and HPLC

Sodium dodecyl sulfate-polyacrylamide gel electrophoresis (SDS-PAGE) was carried out on 0.77-mm-thick slab gels containing a 12% polyacrylamide separating gel and a 5% stacking gel using an electrophoresis cell (Bio-Rad, Hercules, CA, USA) according to the manufacturer’s instructions. Molecular mass marker proteins were from Bio-Rad and Coomassie brilliant blue staining reagents was from Amersham Biosciences. F2 and F1 fractions was freeze-dried and prepared by distilled water to 0.1 mg/mL. Protein concentration was measured by the Bradford method using bovine serum albumin as a standard. The molecular mass of the purified antifungal protein was determined by comparison of its electrophoretic mobility with those of molecular mass marker proteins.

The concentrated F2 sample obtained from Sephadex G-75 column chromatography was filtered through a 0.45-μm polytetrafluoroethylene (PTFE) membrane (Schleicher & Schuell, Keene, NH, USA) and injected onto a Agilent Zorbax 300SB-C_18_ reversed-phase column [150 mm (length) × 4.6 mm (internal diameter), 3.5-μm particle diameter]. The mobile phase consisted of 0.2% trifluoroacetic acid (TFA) in water (solvent A) and 0.2% TFA in acetonitrile (solvent B) using the following gradient program: 0–45 min, 10%–90% B. The flow rate was 2.0 mL/min, and the sample injection volume was 20 μL. The pattern of the eluent was monitored at 280 nm.

### 4.7. Effects of Enzymes, Heat, and pH

The sensitivity of the antifungal protein to enzymes was tested using the purified antifungal protein, which was treated at 37 °C and pH 7.2 for 2 h with trypsin and proteinase K. The final concentration of each enzyme was 1 mg/mL. After incubation, the compounds were assayed for the residual activity using *Rhizoctonia solani* as the indicator strain. To determine the thermal stability by two methods, the purified antifungal protein was treated at pH 7.0 and different temperature (50 °C, 60 °C, 70 °C, 80 °C, 90 °C) for 30 min. After cooling to room temperature, the residual antifungal activity was assayed. To assay the resistance of the purified antifungal protein to pH changes, the pH was adjusted to 2.0, 3.0, 4.0, 5.0, 6.0, 7.0, 8.0, 9.0, 10.0 with 0.1 mol/L HCl and 0.1 mol/L NaOH and kept at room temperature for 30 min. Then each sample was adjusted to pH 7.0 and assayed for the residual activity.

### 4.8. Measurement of Enzyme Activities

For measuring the following several enzyme activities, F2 protein solution was prepared by distilled water to 0.1 mg/mL. Chitinase activity was measured using colloidal chitin as substrate [27]. F2 protein solution (0.5 mL) was added to substrate solution (1.0 mL), which contained 1.3% colloidal chitin in a phosphate buffer (50 mmol/L, pH 7.0). The mixture was then incubated at 37 °C for 10 min. Following centrifugation, the quantity of reducing sugar produced in the supernatant was determined using the method of Imoto and Yagishita [28] using N-acetylglucosamine as a reference compound.

Cellulase xylanase or activity was measured using carboxymethyl cellulose (CMC) or larch wood xylan as substrates. F2 protein solution (0.1 mL) was added to substrate solution (0.4 mL), which contained 0.5% xylan or 1.25% CMC in an acetate buffer solution (125 mmol/L, pH 5.0). Following incubation at 37 °C for 10 min and centrifuging, the amount of reducing sugar produced in the supernatant was measured using the dinitrosalicylic acid (DNS) method. One unit of xylanase or cellulase activity was defined as the quantity of enzyme required to release 1 µmol of reducing sugars per minute at 37 °C and pH 5.0.

To assay protease activity, F2 protein solution (0.2 mL) was mixed with 1% (w/v) casein in phosphate buffer (pH 7.0) (2.5 mL) and incubated at 37 °C for 10 min. The reaction was stopped by the addition of 0.19 mol/L trichloroacetic acid (5 mL). The reaction mixture was then centrifuged and the soluble peptide in the supernatant was measured using tyrosine as the reference compound. One unit of protease activity was defined as the quantity of enzyme required to produce 1 µmol of tyrosine per minute.

Lysozyme activity was determined using a spectrophotometer by measuring the rate of lysis of *M. lysodeikticus* cells. The reaction mixture contained a *M. lysodeikticus* cell suspension (optical density of 1.5, 1.5 mL) in 50 mmol/L phosphate buffer (pH 7.0) and F2 protein solution (1.5 mL). Cell suspensions of *M. lysodeikticus* were prepared as described previously [29]. The mixture was incubated at 37 °C for 30 min with continuous monitoring of the optical density at 660 nm. The control sample contained 1.5 mL of buffer in place of the enzyme. One unit of lysozyme activity was defined as the amount of enzyme required to reduce the optical density at 660 nm by 0.01 per minute [29].

*N*-acetylglucosaminidase was assayed with *p*-nitrophenyl-*N*-acetyl-β-D-glucosaminide (*p*-NP-GlcNAc) as substrate. The reaction mixture contained 5 mmol/L *p*-NP-GlcNAc (0.1 mL) in 50 mmol/L sodium phosphate buffer (pH 7.0) and F2 protein solution (0.1 mL) with a total volume of 0.2 mL. The reaction was stopped by adding 0.25 mol/L Na_2_CO_3_ (2 mL) following incubation for 10 min at 37 °C and the absorbance of the *p*-nitrophenol released was measured at 405 nm. One unit of enzyme activity was defined as the quantity of enzyme required to release 1 µmol of p-nitrophenol per minute at 37 °C.

## 5. Conclusions

In the present study, an antifungal F2 protein from B. *licheniformis* BS-3 was purified and characterized. The purification to homogeneity to F2 protein by mmonium sulfate precipitation, DEAE-52 column chromatography and Sephadex G-75 column chromatography. F2 protein was purified 18.80-fold and recovery was 43.15%. The relative molecule weight of F2 protein was estimated to be 31.0 kDa on SDS-PAGE. F2 protein had better inhibitory effects *on Aspergillus niger, Magnaporthe oryzae, Rhizoctonia solani* and *Fusarium oxysporum* (schl.) f. sp. *benincasae*. Using *Rhizoctonia solani* as the indicator, F2 protein displayed higher antifungal activity, retaining more than 97% activity at temperature below 70 °C and above 90% in pH 6 to pH 10 conditions, the optimal pH was pH 8.0. F2 protein was assayed using various substrates to determine the enzymatic activities, it showed the hydrolyzing activity on casein, however, no enzymatic activities on colloidal chitin, CM-cellulose, xylan, *M. lysodeikticus*, *p*-nitrophenyl-N-acetylglucosaminide. This antifungal protein is expected to develop as a biological prevention agent for farm crops.
